# Lipid rafts: linking prion protein to zinc transport and amyloid-β toxicity in Alzheimer's disease

**DOI:** 10.3389/fcell.2014.00041

**Published:** 2014-08-20

**Authors:** Nicole T. Watt, Heledd H. Griffiths, Nigel M. Hooper

**Affiliations:** ^1^Division of Cardiovascular and Diabetes Research, Multidisciplinary Cardiovascular Research Centre, University of LeedsLeeds, UK; ^2^Faculty of Medical and Human Sciences, Institute of Brain, Behaviour and Mental Health, University of ManchesterManchester, UK

**Keywords:** Alzheimer's disease, AMPA receptor, amyloid, cholesterol, prion protein, zinc

## Abstract

Dysregulation of neuronal zinc homeostasis plays a major role in many processes related to brain aging and neurodegenerative diseases, including Alzheimer's disease (AD). Yet, despite the critical role of zinc in neuronal function, the cellular mechanisms underpinning its homeostatic control are far from clear. We reported that the cellular prion protein (PrP^C^) is involved in the uptake of zinc into neurons. This PrP^C^-mediated zinc influx required the metal-binding octapeptide repeats in PrP^C^ and the presence of the zinc permeable AMPA channel with which PrP^C^ directly interacted. Together with the observation that PrP^C^ is evolutionarily related to the ZIP family of zinc transporters, these studies indicate that PrP^C^ plays a key role in neuronal zinc homeostasis. Therefore, PrP^C^ could contribute to cognitive health and protect against age-related zinc dyshomeostasis but PrP^C^ has also been identified as a receptor for amyloid-β oligomers which accumulate in the brains of those with AD. We propose that the different roles that PrP^C^ has are due to its interaction with different ligands and/or co-receptors in lipid raft-based signaling/transport complexes.

## Zinc homeostasis and alzheimer's disease

Zinc is a trace element that is essential for life and whose importance to the function of the central nervous system is increasingly being appreciated. Zinc serves as a cofactor for >300 enzymes that regulate a variety of cellular processes and signaling pathways, and is also a key structural component of numerous other proteins (Frederickson et al., 2005; Sensi et al., 2009). In the brain, which has one of the highest zinc contents with respect to other organs, zinc-containing axons are particularly abundant in the hippocampus and cortex (Toth, 2011). Zinc is predominantly, but not exclusively, localized within synaptic vesicles at glutamatergic nerve terminals [sometimes referred to as gluzinergic neurons (Mocchegiani et al., 2005)]. During neuronal activity, zinc is released along with glutamate into the synaptic cleft where it affects the activity of various receptors. In addition, zinc is itself a signaling molecule, and within the neuron it regulates the activity of multiple enzymes and plays a critical role in the formation and stabilization of the postsynaptic density (Beyersmann and Haase, 2001; Grabrucker et al., 2011; Wilson et al., 2012). Both intracellular and extracellular zinc concentrations must be tightly maintained within narrow optimal ranges for the correct functioning of the nervous system. An excess influx of zinc can damage postsynaptic neurons (Plum et al., 2010) and zinc deficiency affects neurogenesis and increases neuronal apoptosis, which can lead to learning and memory deficits (Szewczyk, 2013). Under normal circumstances, zinc homeostasis is maintained by the coordinated actions of a range of different proteins involved in its uptake, efflux, and intracellular storage and trafficking. Zinc enters neurons through members of the ZIP (Zrt/Irt-like Protein; SLC39) family of zinc transporters, as well as through activated voltage-gated Ca^2+^ channels, α-amino-3-hydroxy-5-methyl-4-isoxazoleproprionate (AMPA) receptors, and N-methyl-D-aspartate (NMDA) receptors (Cousins et al., 2006; Sensi et al., 2009). Zinc exporters, members of the zinc transporter (ZnT; SLC30) family transport zinc from the cytosol to the lumen of intracellular organelles or out of the cell (Sensi et al., 2009).

Alzheimer's disease (AD) is the most prevalent form of dementia, affecting millions of individuals world-wide. With currently no cure, the economic and social costs associated with the disease are set to increase dramatically with our aging population. AD is characterized by the deposition in the brain of extracellular plaques of amyloid-β (Aβ) peptide and intracellular inclusions of tau protein. Aβ is proteolytically cleaved from the larger amyloid precursor protein (APP), and both Aβ and APP have binding sites for zinc (Watt et al., 2010; Wong et al., 2014). Zinc, particularly that released from glutamatergic nerve terminals, has a crucial role to play in the aggregation of Aβ into neurotoxic oligomers and fibrils (Bush et al., 1994; Esler et al., 1996; Deshpande et al., 2009), and is also co-localized with Aβ in amyloid plaques (Dong et al., 2003). The activity of the zinc transporter ZnT-3 is required for the transport of zinc into the glutamate-containing presynaptic vesicles. Key evidence for a link between zinc and amyloid pathology in AD comes from the crossing of ZnT-3 knockout mice with Tg2576 mice, a commonly used transgenic mouse model of AD. The crossed mice had minimal synaptic zinc and as a consequence both brain plaque load and amyloid angiopathy were significantly reduced (Lee et al., 2002; Friedlich et al., 2004). Furthermore, in a separate study of aged ZnT-3 knockout mice, there were marked differences in learning and memory observed compared to wild type mice, supporting a requirement for zinc in memory function and the maintenance of synaptic health upon aging (Adlard et al., 2010).

## PrP^C^ and neuronal zinc uptake

Recently we reported that PrP^C^ facilitates the uptake of zinc into neuronal cells (Watt et al., 2012). PrP^C^ is infamous because of its conformational conversion into PrP^Sc^ being responsible for the fatal transmissible spongiform encephalopathies, such as Creutzfeldt-Jakob disease. PrP^C^ is a glycosyl-phosphatidylinositol (GPI)-anchored protein located on the surface of neurons, at both pre- and post-synaptic sites. It is found throughout the central nervous system and is particularly abundant in the hippocampus and frontal cortex (Sales et al., 1998). Using two zinc specific fluorescent dyes (Newport green and Zinpyr-1) we showed that PrP^C^ enhanced the uptake of zinc into human SH-SY5Y neuroblastoma cells and rat primary hippocampal neurons (Watt et al., 2012). This PrP^C^-mediated zinc influx required the metal-binding octapeptide repeat region in PrP^C^ but not its endocytosis. We then used selective channel antagonists to identify that AMPA receptors were involved in the PrP^C^-mediated zinc uptake, and that PrP^C^ interacted with both GluA1 and GluA2 AMPA receptor subunits as shown by co-immunoprecipitation. Intracellular protein tyrosine phosphatase activity, which is potently inhibited by zinc (Brautigan et al., 1981; Wilson et al., 2012), was increased in the brains of PrP^C^ null mice, providing evidence of a physiological consequence of altered zinc uptake in the absence of PrP^C^. These observations provided the first mechanistic explanation for the reduced zinc in the hippocampus and other brain regions of PrP^C^ null mice (Pushie et al., 2011) and indicate that PrP^C^ is a key player in neuronal zinc uptake.

**Figure 1:**
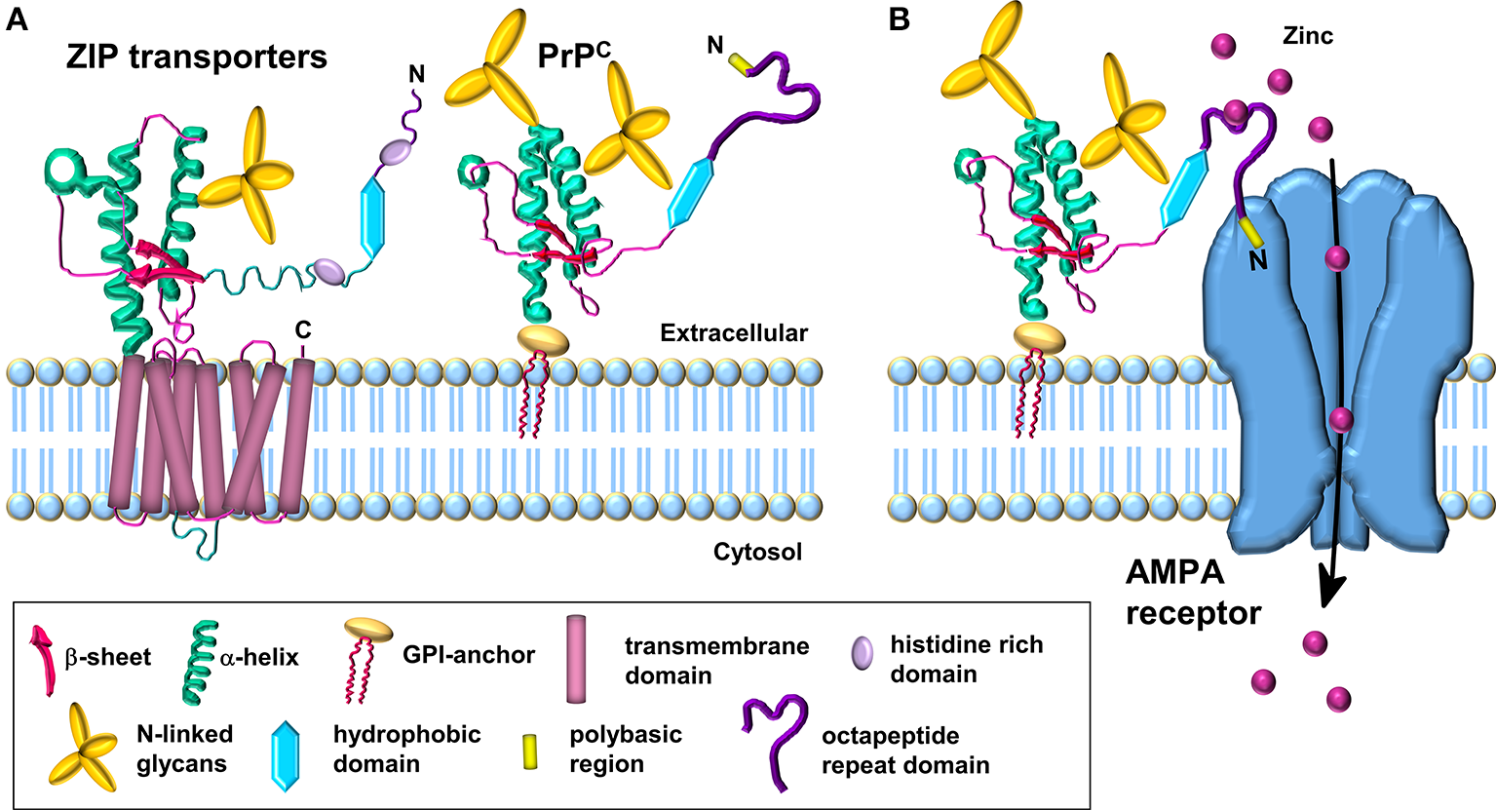
**Schematic comparison of ZIP transporters and the PrP^C^-AMPA receptor zinc transporter. (A)** Model of the LIV-1 subfamily of ZIPs with a prion-like ectodomain which is coupled to the C-terminal transmembrane domain. PrP^C^ is structurally similar to the ectodomain of the ZIP transporter. **(B)** PrP^C^ acts as a sensor for zinc in the extracellular space and coordinates the low affinity binding of zinc to the octapeptide repeats. The N-terminal polybasic region of PrP^C^ interacts with the AMPA receptor subunits; this interaction facilitates the transport of zinc through the AMPA receptor which forms a channel across the membrane for the uptake of zinc in a manner similar to the C-terminal region of the ZIP transporter. Modified from Watt et al. (2013).

Interestingly, during the course of our work showing that PrP^C^ mediates neuronal zinc uptake, the protein was reported to be evolutionarily related to a subset of the ZIP family of zinc transporters (Schmitt-Ulms et al., 2009). Bioinformatic analysis revealed that the N-terminal extracellular domain of a distinct sub-branch of the LIV-1 subfamily of ZIPs that includes ZIPs 5, 6, and 10 had sequence similarity to the C-terminal globular domain of PrP^C^. These three ZIPs are also equipped with histidine-rich sequences N-terminal to their “prion-like” domain, capable of divalent metal binding, which is reminiscent of the octapeptide repeat domain of PrP^C^ (Figure 1A). Interestingly, the orientation and distance of the “prion-like” domain to the respective membrane attachment sites in both PrP^C^ and the ZIPs are similar (Figure 1A), and the primary sequence of the first transmembrane domain in the ZIPs and the GPI anchor attachment sequence of PrP^C^ are also comparable, providing further evidence that PrP^C^ is evolutionarily related to members of the ZIP family (Schmitt-Ulms et al., 2009).

As PrP^C^ is a GPI-anchored protein residing in the outer leaflet of the plasma membrane, in order to correlate a function for PrP^C^ in the transport of zinc across the lipid bilayer, we proposed that PrP^C^ requires the assistance of the channel properties of another protein(s), such as AMPA receptors, in order to achieve zinc transport (Figure 1B) (Watt et al., 2012). The octapeptide repeats in the GPI-anchored PrP^C^ act as a zinc sensor/scavenger in the extracellular environment which then presents the metal to the transmembrane channel for uptake of the zinc into the cell (Watt et al., 2013). Interestingly, not all ZIP family members have an extracellular N-terminal extension that may function in zinc sensing: for example ZIPs 1, 2, 3, 9, and 11 have minimal N-terminal extensions on the first transmembrane domain. This raises the intriguing possibility that the two properties (zinc sensing and zinc channel) present together in certain ZIPs (e.g., ZIPs 6 and 10) reside in separate proteins in those ZIPs with minimal N-terminal extensions. It will be interesting to see if PrP^C^ acts as the zinc sensor in combination with certain ZIPs, similar to the situation we have proposed for PrP^C^ and the AMPA receptor (Figure 1B) (Watt et al., 2012, 2013). Such PrP^C^-based zinc sensing modules may be restricted to higher eukaryotes in which PrP^C^ with the octapeptide metal binding repeats is present.

In addition to its role in zinc metabolism, PrP^C^ also plays a role in the homeostasis of other metals such as iron and copper (Kozlowski et al., 2010; Singh et al., 2014). PrP^C^ expression alters regional zinc, copper and iron content in the mouse brain (Pushie et al., 2011) and there appears to be crosstalk between the different metals, with zinc modulating copper coordination to the octapeptide repeats (Stellato et al., 2011). In addition, PrP^C^ modulates NMDA receptor activity in a copper-dependent manner (Stys et al., 2012), suggesting that multiple receptors/channels may be regulated by PrP^C^ in a ligand/metal-dependent manner.

## A cell-surface, lipid raft-based complex involved in the regulation of zinc uptake

The GPI-anchored PrP^C^ is localized in cholesterol-rich, detergent-resistant lipid rafts at the cell surface (Taylor et al., 2005) and it has been proposed that PrP^C^ functions as a key scaffolding protein for the dynamic assembly of cell surface signaling modules (Linden et al., 2008). PrP^C^, along with the microdomain-forming flotillin or caveolin proteins, may lead to the local assembly of membrane protein complexes at sites involved in cellular communication, such as cell-cell contacts, focal adhesions, the T-cell cap and synapses (Solis et al., 2010). Lipid rafts are essential for synapse development, stabilization and maintenance and caveolin-1 organizes and targets synaptic components to rafts (Hering et al., 2003; Willmann et al., 2006; Guirland and Zheng, 2007). Interestingly, when ZIP1 and ZIP3 were stably expressed in HEK293 cells, the punctate cell surface staining observed led the authors to suggest that they were localized to lipid rafts (although no experimental evidence for this was provided) (Wang et al., 2004). Furthermore, treatment of the HEK293 cells with methyl-β-cyclodextrin, which sequesters cholesterol and disrupts cholesterol-rich lipid rafts, resulted in a more diffuse surface staining (Wang et al., 2004), reminiscent of what is observed for PrP^C^ and other GPI-anchored proteins (Parkin et al., 2003; Taylor et al., 2005). We also have disrupted rafts in neuronal cells using methyl-β-cyclodextrin and observed a reduction in zinc uptake, an effect exacerbated when the cells also expressed PrP^C^ (Watt and Hooper, unpublished). In contrast, it has been reported that ZIP10 only partially colocalizes with PrP^C^ in N2a cells and is not detected in detergent-resistant rafts (Ehsani et al., 2012). These data raise the intriguing possibility that a cell-surface, lipid raft-based complex, possibly stabilized by PrP^C^, regulates the cell surface expression of certain zinc transporters and thus zinc uptake.

## PrP^C^ is a cell surface receptor for Aβ oligomers

In 2009, PrP^C^ was identified as a high-affinity receptor for Aβ oligomers, the primary neurotoxic species in AD (Lauren et al., 2009). The presence of PrP^C^ in hippocampal slices was shown to be responsible for the Aβ oligomer-mediated inhibition of long-term potentiation (LTP) (Lauren et al., 2009). PrP^C^ was also required for the manifestation of memory impairments in an AD mouse model (Gimbel et al., 2010), which were reversed by intra-cerebral infusion of an anti-PrP^C^ monoclonal antibody (Chung et al., 2010). Immuno-targeting of PrP^C^ was shown to block completely the LTP impairments caused by Aβ oligomers derived from human AD brain extracts (Barry et al., 2011; Freir et al., 2011). Aβ oligomers bound to PrP^C^ activate the non-receptor tyrosine kinase Fyn (Um et al., 2012; Rushworth et al., 2013) and results in pathological changes in tau (Larson et al., 2012). Although there is general consensus that PrP^C^ can bind oligomeric forms of Aβ, some studies dispute a role for PrP^C^ in mediating Aβ toxicity (Calella et al., 2010; Kessels et al., 2010).

**Figure 2:**
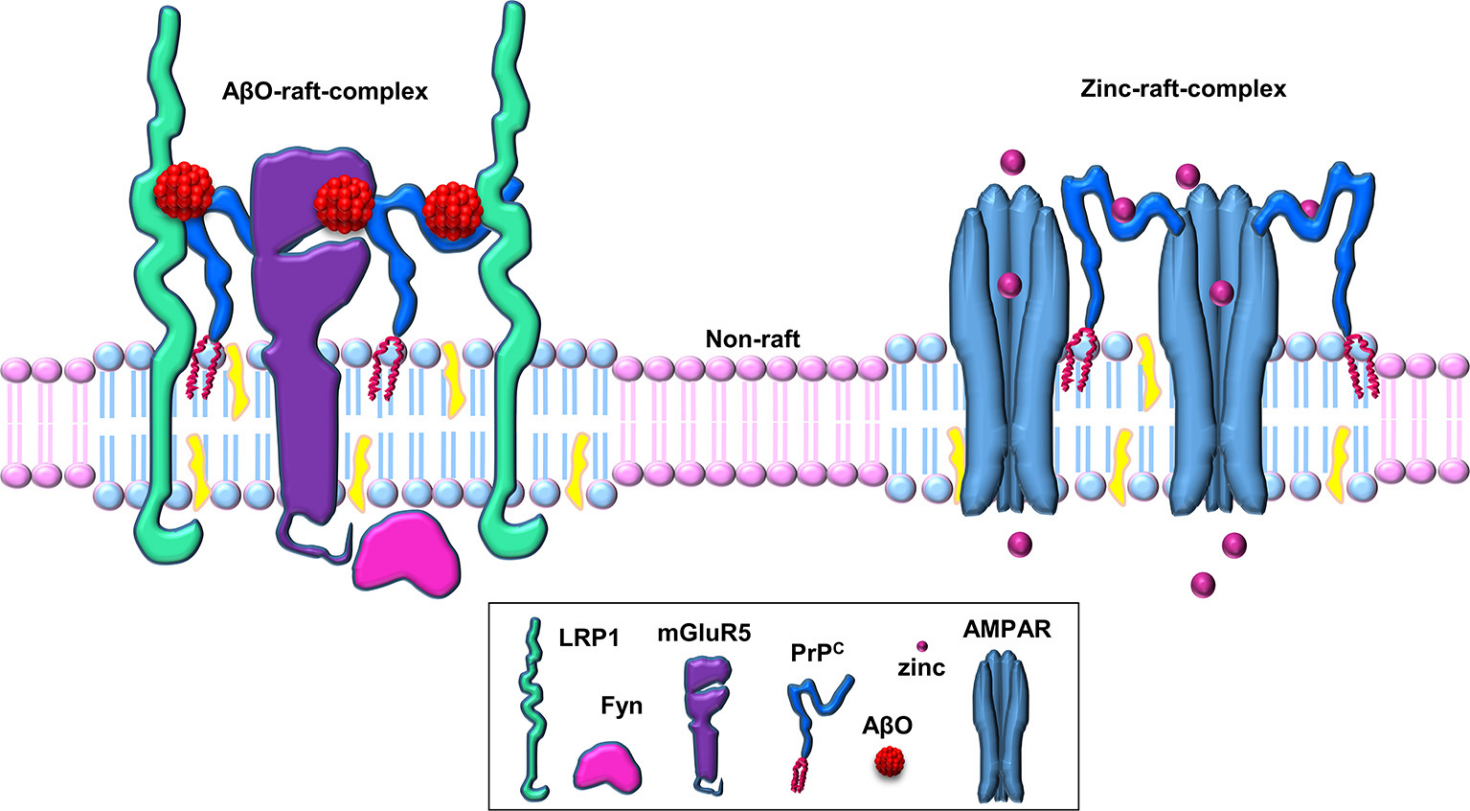
**Schematic of PrP^C^-based cell-surface, raft-based complexes involved in zinc uptake and Aβ binding**. PrP^C^ acts as a hub for cell surface, lipid raft-based signaling/transport complexes. PrP^C^ associates with various transmembrane proteins (such as AMPA receptors, LRP1, mGluR5, etc.) in these multi-protein complexes in a ligand-dependent manner (here shown for the ligands zinc and Aβ oligomers within separate raft-based complexes).

It has been hypothesized that a putative transmembrane co-receptor is required to connect the binding of Aβ to the GPI-anchored PrP^C^ on the outer surface of the plasma membrane with downstream effects inside the cell (Cisse and Mucke, 2009; Lauren et al., 2009). The transmembrane low-density lipoprotein-receptor related protein-1 (LRP1) is highly expressed in neuronal cells (Nykjaer and Willnow, 2002), facilitates the endocytosis of PrP^C^ (Taylor and Hooper, 2007) and has been implicated in the neuronal uptake of Aβ (Fuentealba et al., 2010; Kanekiyo et al., 2011). We hypothesized, therefore, that LRP1 may play a role in the PrP^C^-mediated action of Aβ oligomers (Figure 2). We showed that LRP1 is required for the binding of Aβ oligomers to cells, as well as for their subsequent internalization and cytotoxicity (Rushworth et al., 2013). In parallel with our work on LRP1, Strittmatter and colleagues identified the metabotropic glutamate receptor, mGluR5, as a co-receptor with PrP^C^ for Aβ oligomers and was required to activate Fyn (Um et al., 2013). Furthermore, antagonists of mGluR5 reversed the deficits in learning, memory and synapse density in AD transgenic mice (Um et al., 2013). In addition, the Aβ-PrP^C^-mGluR5 interplay is involved in mediating both long-term depression facilitation and LTP inhibition (Hu et al., 2014). Thus, both LRP1 and mGluR5 appear to be transmembrane co-receptors in the Aβ-PrP^C^ interaction, which are required for the PrP^C^-mediated action of Aβ oligomers. Whether both LRP1 and mGluR5 reside in the same complex with PrP^C^ or in separate signaling complexes is currently unclear.

## A cell-surface, lipid raft-based signaling complex involved in Aβ oligomer action

Cell surface cholesterol-rich, detergent-resistant lipid rafts are intimately involved in the production, aggregation and toxicity of Aβ (Rushworth and Hooper, 2010; Di Paolo and Kim, 2011). For example, these membrane microdomains have been linked with Aβ toxicity via Fyn, and rafts are involved in the neuronal internalization of Aβ (Williamson et al., 2008; Lai and McLaurin, 2010). We investigated whether the integrity of lipid rafts is required for the binding of Aβ oligomers and the subsequent activation of Fyn. Treatment of cells with methyl-β-cyclodextrin caused the re-localization of PrP^C^ and Fyn from detergent-resistant rafts to detergent-soluble, non-raft regions of the membrane as analyzed by sucrose density gradient centrifugation in the presence of Triton X-100. Surprisingly, disruption of the rafts with methyl-β-cyclodextrin significantly reduced (by 80.6%) the cell surface binding of the Aβ oligomers, even though the cell surface expression of PrP^C^ was unaffected, and prevented the Aβ oligomers from activating Fyn (Rushworth et al., 2013). Thus, raft localization of PrP^C^ is required for the binding of Aβ oligomers, and the integrity of rafts and/or other raft-localized proteins are required for the Aβ-mediated activation of Fyn. These observations suggest that there is a cell-surface, raft-based signaling complex that is key to the binding of Aβ oligomers and the subsequent generation of cellular responses.

Aβ oligomers caused mGluR5 receptors to manifest reduced lateral diffusion as they became aberrantly clustered (Renner et al., 2010). Antibodies against PrP^C^ and the NR1 subunit of NMDA receptors had a similar inhibitory effect on Aβ oligomer binding as the antibody against mGluR5, but there was not an additive effect of the individual antibodies, suggesting that mGluR5, PrP^C^, and NR1 may be in proximity to each other as well as the Aβ oligomer binding site (Renner et al., 2010). Interestingly, addition of Aβ oligomers to neurons caused a large increase of mGluR5 in the Triton-resistant fraction (Renner et al., 2010), although no link to its possible redistribution into detergent-resistant lipid rafts was made. In another study, disruption of lipid rafts by cholesterol depletion reduced the interaction of Aβ with α-7-nicotinic acetylcholine receptors (Khan et al., 2010). These observations are consistent with multiple (co)receptor proteins for Aβ oligomers residing in cell-surface, raft-based complex(es).

## PrP^C^ and rafts are dysregulated in aging

Zinc homeostasis plays a major role in many processes related to brain aging. For example, mouse models of accelerated aging, such as senescence-accelerated mouse prone 10 (SAMP10) mice display a low total zinc concentration in synaptic vesicles that is associated with brain atrophy and defects in learning and memory (Saito et al., 2000), and dietary zinc deficiency influences hippocampal learning and memory in an age-dependent manner (Mocchegiani et al., 2005; Szewczyk, 2013). ZIP6 expression has been reported to be decreased in an age-dependent manner (Wong et al., 2013), although nothing has been reported about the expression, subcellular localization or function of any other ZIP in the aged brain. Caveolin-1 has been identified as a novel control point for healthy neuronal aging, with the localization of caveolin-1, PSD95, and AMPA receptors in lipid rafts being decreased in aged (>18 months) mice compared with young (3–6 month) mice (Head et al., 2010). Furthermore, in an aging series (age 20–88 years) of human brains we reported that PrP^C^ was reduced in the hippocampus with increasing age (Whitehouse et al., 2010). Preliminary data indicate that there is a significant reduction of both ZIP1 and ZIP3 in the hippocampus of old (79–88 years) compared with young (20–26 years) individuals (Watt and Hooper, unpublished). These observations suggest that alterations to the structure and function of the cell-surface, raft-based zinc transporter complex may occur in aging and contribute to the age-related dysregulated zinc homeostasis.

Aging is the greatest risk factor for AD and recently the REST protein was reported to have a central role in protecting aging neurons from degeneration (Lu et al., 2014). There is a close relationship between lipid rafts, cholesterol, and the age-associated decline and dysregulation of cellular signaling pathways (Ohno-Iwashita et al., 2010). A key component of a subset of rafts, caveolin-1, was identified as a novel control point for both Aβ-based neurodegeneration and healthy neuronal aging (Head et al., 2010), and caveolin-1 expression is altered in AD (Gaudreault et al., 2004). In addition, the level of PrP^C^ is altered in both the AD and aging brain (Whitehouse et al., 2010, 2013; Larson et al., 2012). Thus, dysregulation of the structure and function of cell-surface, raft-based signaling complexes may occur in, and contribute to, both AD and aging.

## Conclusion

PrP^C^ appears to be involved in multiple physiological and pathological processes, including as highlighted here, neuronal zinc uptake and Aβ oligomer binding and toxicity. We propose that PrP^C^ acts as a hub for cell surface, lipid raft-based signaling/transport complexes, and that the different roles that the protein has are due to the selective interaction of PrP^C^ with different ligands (such as zinc or Aβ oligomers) and/or co-receptors (such as AMPA receptors, LRP1, mGluR5) in these multi-protein complexes (Figure 2). It is likely that these complexes are relatively transient in nature, being stabilized upon binding of a particular ligand for a limited time period before dissociation or endocytosis terminates the signaling or transport process. The use of more sophisticated techniques, such as super resolution light microscopy, should enable the molecular details of these different lipid raft-based signaling/transport complexes to be determined and provide a clearer picture of the role of PrP^C^ and lipid rafts in neuronal zinc transport and Aβ action in AD. It will also be interesting to determine how the interplay between PrP^C^, zinc, and Aβ may underlie aging and age-related diseases.

### Conflict of interest statement

The authors declare that the research was conducted in the absence of any commercial or financial relationships that could be construed as a potential conflict of interest.
